# Investigation of the Effect of Supersonic Flow of Dissociated Nitrogen on ZrB_2_–HfB_2_–SiC Ceramics Doped with 10 vol.% Carbon Nanotubes

**DOI:** 10.3390/ma15238507

**Published:** 2022-11-29

**Authors:** Elizaveta P. Simonenko, Nikolay P. Simonenko, Anatoly F. Kolesnikov, Aleksey V. Chaplygin, Anton S. Lysenkov, Ilya A. Nagornov, Artem S. Mokrushin, Nikolay T. Kuznetsov

**Affiliations:** 1 Kurnakov Institute of General and Inorganic Chemistry of the Russian Academy of Sciences, Leninsky pr., 31, 119991 Moscow, Russia; 2 Ishlinsky Institute for Problems in Mechanics of the Russian Academy of Sciences, 101-1 pr. Vernadskogo, 119526 Moscow, Russia; 3 A.A.Baikov Institute of Metallurgy and Materials Science, Russian Academy of Sciences, Leninskii pr. 49, 119334 Moskow, Russia

**Keywords:** UHTC, carbon nanotubes, CNT, borides, SiC, induction plasmatron

## Abstract

The method of fabricating dense ultra-high temperature ceramic materials ZrB_2_–HfB_2_–SiC–C_CNT_ was developed using a combination of sol-gel synthesis and reaction hot pressing approaches at 1800 °C. It was found that the introduction of multilayer nanotubes (10 vol.%) led to an increase in the consolidation efficiency of ceramics (at temperatures > 1600 °C). The obtained ZrB_2_–HfB_2_–SiC and ZrB_2_–HfB_2_–SiC–C_CNT_ materials were characterized by a complex of physical and chemical analysis methods. A study of the effects on the modified sample ZrB_2_–HfB_2_–SiC–C_CNT_ composition speed flow of partially dissociated nitrogen, using a high-frequency plasmatron, showed that, despite the relatively low temperature established on the surface (≤1585 °C), there was a significant change in the chemical composition and surface microstructure: in the near-surface layer, zirconium–hafnium carbonitride, amorphous boron nitride, and carbon were present. The latter caused changes in crucial characteristics such as the emission coefficient and surface catalyticity.

## 1. Introduction

Ceramic materials based on zirconium and hafnium diborides, modified with silicon carbide, are of great interest to the scientific community as promising options for use in extreme operating conditions–under high mechanical and thermal loads, including conditions involving aerodynamic heating to temperatures >2000 °C by high-speed air flows [1,2,3,4,5,6]. This is due to the high melting temperatures of both the initial components ZrB_2_, HfB_2_, and SiC and their oxidation products ZrO_2_ and HfO_2_, the increased thermal conductivity of metal diborides, as well as the good mechanical properties of the materials (in particular, high hardness and strength). The optical properties also allow the materials on which they are based to be considered as components of alternative energy devices [7,8,9,10,11]. The formation of a protective borosilicate glass layer during oxidation, which prevents rapid degradation of deeper ceramic layers, leads to the fact that materials based on zirconium/hafnium diborides and silicon carbide can withstand even long-term exposure to high-speed dissociated air flows at surface temperatures of up to 2500 °C [12,13,14,15,16,17,18,19,20].

Nevertheless, ultra-high-temperature ceramic materials (UHTC) of ZrB_2_(HfB_2_)–SiC composition have their own drawbacks: in particular, they are characterized by low fracture resistance and heat resistance, which significantly limits the prospects of their application in nodes requiring high reliability, as well as involving cyclic heating with large drops in temperature.

One way to improve material properties is to use solid solutions based on these phases, instead of individual zirconium or hafnium diborides, including so-called high-entropic compositions [21,22,23]. In this aspect, in [24] it was found that the introduction of HfB_2_ improved the free sintering at temperatures of 2100–2150 °C of ZrB_2_–SiC ceramic material (in the presence of B_4_C and MoSi_2_); in this case, a solid solution (Zr,Hf)B_2_ is formed. In the paper [25], it was shown that partial substitution of ZrB_2_ by hafnium diboride in MB_2_–30 vol.% SiC ceramics (HfB_2_ content varied from 4 to 12 vol.%) obtained by spark plasma sintering at temperatures of 1650–1800 °C and pressures of 20–40 MPa (holding time 4–14 min) led to higher oxidation resistance when samples were exposed to air in a furnace at 1400 °C.

The introduction of other modifiers from a number of refractory substances, such as carbides [22,26,27] and metal nitrides [28,29,30], as well as carbon materials [31,32,33,34], also has a significant influence on the mechanical characteristics of UHTCs. The use of carbon nanotubes (CNTs) as dopants is of high interest [35,36,37,38,39,40,41,42,43,44,45,46,47]. In general, it is shown that such modification makes it possible to significantly increase the fracture resistance of materials (up to K_IC_ = 6–10 MPa·m^1/2^), but the process of making such ceramic composites requires the choice of hot-pressing temperature and maximum disaggregation of CNTs and their dispersion in the volume of materials. 

In the literature, the influence of the introduction of CNTs on the mechanical characteristics of UHTC materials ZrB_2_(HfB_2_)–SiC is mainly considered, while the study of the oxidation features of similar composites or their behaviour in high-enthalpy jets of other gases is found in studies [38,40,44,46]. So, for the ceramic materials in the systems ZrB_2_+SiC, ZrB_2_+HfB_2_+SiC, and ZrB_2_+HfB_2_+SiC+CNT (6 vol.% CNT) obtained by the SPS (1850 °C, 30 MPa, holding time 10 min) method [38], the thermal behaviour at heating to 1200 °C in air current (20 mL/min) was studied: for doped CNT, the greatest increase in mass was fixed by oxidation. In the same paper [42], it is shown that the exposure of samples ZrB_2_–20 vol.% SiC–10 vol.% C_CNT_ to air current (T_max_ = 2750 °C) for 30 s using a plasma arc unit did not lead to changes in the appearance, and CNTs using Raman spectroscopy were found both in the non-oxidized region and in the oxidized layer based mainly on zirconium and silicon oxides. 

Thus, ultra-high-temperature ceramic materials ZrB_2_(HfB_2_)–SiC modified with carbon nanotubes are extremely promising for use in modern models of aerospace technology, ceramic propulsion systems and components of alternative energy devices. 

The authors have not found a study of the interaction of ultra-high-temperature ZrB_2_+HfB_2_+SiC+C_CNT_ ceramic with high-enthalpy flows of other gas compositions, such as nitrogen, which is the main component of the Saturn satellite Titan atmosphere (98.4%) [47,48], or carbon dioxide, prevailing in the Mars atmosphere [49,50,51,52], despite the extreme practical relevance. The authors previously studied the behaviour in supersonic jets of dissociated nitrogen of graphite samples [53] and HfB_2_–SiC and HfB_2_–SiC–C_(graphene)_ ceramics [54], which showed that there occurred a significant change in the surface chemical nature due to ongoing reactions with atomic nitrogen and silicon removal at elevated temperatures, which causes a change in the emissivity and catalyticity.

The aim of this work is to develop a method for obtaining the ultra-high-temperature ZrB_2_–HfB_2_–SiC ceramic material and its modification with 10 vol.% carbon multilayer nanotubes and to study the behaviour of the latter in a supersonic flow of dissociated nitrogen.

## 2. Materials and Methods

Reagents used: Tetraethoxysilane (TEOS) Si(OC_2_H_5_)_4_ (>99.99%, EKOS-1 JSC, Moscow, Russia), LBS-1 bakelite varnish (Karbolit OJSC, Moscow, Russia), formic acid CH_2_O_2_ (>99%, Spektr-Chem LLC, Moscow, Russia), hafnium diboride (>98%, particle size 2–3 microns, aggregate size ~20–60 microns, Tugoplavkie Materialy LLC, Russia), zirconium diboride (>98%, MP Complex LLC, Moscow, Russia), multi-walled CNTs (brand “Dealcom”, SPE “Center of Nanotechnologies”, Moscow, Russia).

The synthesis of the composite powders required for the subsequent reaction hot pressing was carried out by the method similar to those presented in [12,55], in which the UHTC material HfB_2_–SiC was modified by the carbon nanomaterial and reduced graphene oxide was obtained. In particular, powders of ZrB_2_, HfB_2_ and CNTs were dispersed in the solution of phenol-formaldehyde resin LBS-1, then tetraethoxysilane was introduced under stirring and subjected to controlled hydrolysis (ratio *n*(Si):*n*(H_2_O) was 1:5). After gelling and step drying, the xerogels were carbonized in a dynamic vacuum at 400 °C for 2 h, resulting in composite ZrB_2_–HfB_2_–SiO_2_–C_(CNT+amorphous carbon)_ powders. The molar ratio of the formed silicon dioxide and amorphous carbon, obtained as a result of phenol formaldehyde resin pyrolysis, was 1:3.05, which is necessary for the full course of the carbothermic synthesis of nanocrystalline silicon carbide in the consolidation of ceramics.

To produce 35 vol.% ZrB_2_–35 vol.% HfB_2_–30 vol.% SiC (ZHS) and 31.5 vol.% ZrB_2_–31.5 vol.% HfB_2_–27 vol.% SiC–10 vol.% C_CNT_ (ZHSC) ceramic materials, synthesized composite powders were sintered in graphite moulds using a hot-pressing press by Thermal Technology Inc. (model HP20-3560-20) in argon current at 1800 °C (heating rate 10 °C/min, holding time 30 min) and uniaxial pressure 30 MPa [12,15,55,56,57,58]. 

The X-ray patterns of the obtained ceramic materials ZHS and ZHSC, as well as the surface of the ZHSC sample after exposure to a supersonic flow of dissociated nitrogen, were recorded on a Bruker D8 Advance X-ray diffractometer (CuK_α_ radiation, resolution 0.02° with signal accumulation in the point for 0.3 s). X-ray phase analysis was performed using the program MATCH!–Phase Identification from Powder Diffraction, Version 3.8.0.137 (Crystal Impact, Germany), Crystallography Open Database (COD).

Raman spectra were recorded on a Renishaw InVia Reflex Raman Spectrometer (50 × objective, ~2 μm pad diameter) using a He-Ne laser (633 nm) with a power of <2.8 mW.

The study of the resistance of the obtained material to the supersonic flow of underexpanded high-enthalpy nitrogen jets, flowing from a water-cooled conical nozzle with an outlet cross-section diameter of 50 mm, was carried out on a 100 kW high-frequency induction plasmatron VGU-4. The distance from the nozzle to the sample was 30 mm, the nitrogen flow rate was 3.6 g/s, and the pressure in the chamber was in the range of 8.6–9.3 × 10^2^ Pa. The sample in the form of a cylinder, with a diameter of 15 mm and a thickness of ~3.2 mm, was injected into the high enthalpy jet at the plasmatron anode power (N) of 64 kW, and then the sample was incubated at these conditions for 10 min (600 s). The geometry of the model, in which the samples were fixed, is described in detail in [53,54,59]: the samples were mounted on friction in the seat of a water-cooled calorimeter, the gap was filled with flexible SiC-based thermal insulation and carbon fibres in order to minimize heat losses. 

The measurement of the average surface temperature of the heated sample in its central area (diameter of sighting area was ~5 mm) was performed using a Mikron M-770S infrared pyrometer in spectral ratio pyrometer mode (temperature interval 1000–3000 °C). Temperature distribution over the sample surface was studied using a Tandem VS-415U thermovisor. The thermal images were recorded at the set value of the spectral coefficient of radiation ε at the wavelength 0.9 µm equal to 1, since in the course of exposure it was supposed to change ε. A correction made to the real values of average temperatures in the central region, determined with an infrared pyrometer, made it possible to estimate the spectral coefficient of radiation and its change during the course of exposure. The method of determining the heat flux to the face of the sample is described in detail in [54]. 

A study of the surface microstructure features of the obtained materials before and after exposure to a supersonic flow of dissociated nitrogen was carried out by scanning electron microscopy (SEM) on a three-beam NVision 40, Carl Zeiss workstation with an accelerating voltage of 1, 2 and 20 kV. The elemental composition of the regions was determined using an EDX Oxford Instruments energy dispersive analysis device.

The evaluation of possible reactions between the components of ZrB_2_–HfB_2_-SiC-C UHTCs and molecular and atomic nitrogen was performed using the IVTANTERMO software package with a built-in database at standard conditions [60,61]. ZrB_2_ was chosen as a model diboride phase, the most probable reactions of molecular and atomic nitrogen involving ZrB_2_ and SiC were analysed.

## 3. Results and Discussion

### 3.1. Obtaining ZHS and ZHSC Ceramic Materials

**Figure 1:**
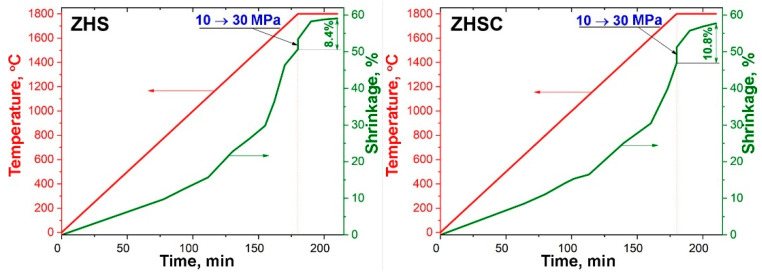
Shrinkage curves as a function of temperature and hot-pressing pressure for ZHS (**left**) and ZHSC (**right**) samples.

As can be seen in Figure 1, for both samples, despite the fact that the initially applied pressure was only 10 MPa, significant shrinkage began already at 1100–1200 °C, which may be associated with easier sliding of grains relative to each other due to the presence of softened SiO_2_. A sharp increase in the rate of consolidation was observed as the temperature reached 1550–1600 °C, which may be due to the ongoing synthesis of silicon carbide. It should be noted that for the ZHSC sample, the bulk density of the initial powder was noticeably higher, which affected the value of total shrinkage. Nevertheless, at the final stage of the reaction hot pressing, after reaching the target values of 1800 °C temperature and 30 MPa pressure, the effect of the introduction of multi-walled CNTs was significantly reflected in the consolidation efficiency: during extraction, the shrinkage value for the modified ZHSC sample was 10.8% compared to 8.4% for the ZHS sample.

The density of the samples was 7.11 ± 0.12 g/cm^3^ for ZHS and 7.04 ± 0.06 g/cm^3^ for ZHSC. The relative density for them is 101 and 108%, respectively, based on the calculated value obtained by the additive method (densities are taken for ZrB_2_ 6.2 [62], HfB_2_ 11.2 [63], SiC 3.2 g/cm^3^ [64], for CNTs a value of 1.8 g/cm^3^ was taken). The excess relative density of 100% may be due to the reaction between zirconium and hafnium oxide impurities at the ZrB_2_ and HfB_2_ particles boundaries during a consolidation with the formation of corresponding carbides at the interfaces, as well as the too low accepted density value of CNTs.

**Figure 2:**
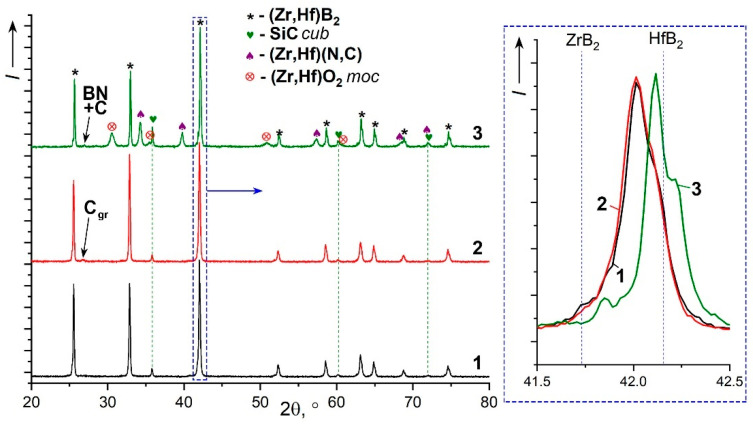
X-ray diffraction patterns of ZHS (1), ZHSC (2), and the surface of the ZHSC sample after exposure to a supersonic flow of dissociated nitrogen (3); the inset area in the range 2θ = 41.5–42.5°, in which the position of the maximum intensity reflexes of ZrB_2_ [66] and HfB_2_ [67].

The data of X-ray phase analysis (Figure 2, X-ray pattern 1 and 2) indicate the synthesis of cubic silicon carbide [65]. No traces of crystalline SiO_2_ were found. The position of the reflexes of maximum intensity indicates the formation of a solid solution of (Zr,Hf)B_2_ instead of separate hexagonal phases ZrB_2_ [66] and HfB_2_ [67]. The performed full-profile analysis using the TOPAS software made it possible to calculate the average crystal lattice parameters, which indicate the formation of a solid solution of the tentative Zr_0.31(4)_Hf_0.69(4)_B_2_ composition for both ZHS and ZHSC samples. Some asymmetry of the diboride phase reflexes (Figure 2, inset) testifies that in addition to this composition, some grains with elevated zirconium content can be present in the samples. No impurities of crystalline cubic ZrC and HfC carbide phases were detected. For the sample ZHSC (Figure 2, X-ray pattern 2) the presence of low intervisibility reflex, which can be attributed to the trigonal graphite phase [68], is noted. Obviously, this is due to the introduced multi-walled CNTs, for which the formation of aggregates or some graphitization of nanotube packages during consolidation at elevated temperatures (1800 °C) is possible.

**Figure 3:**
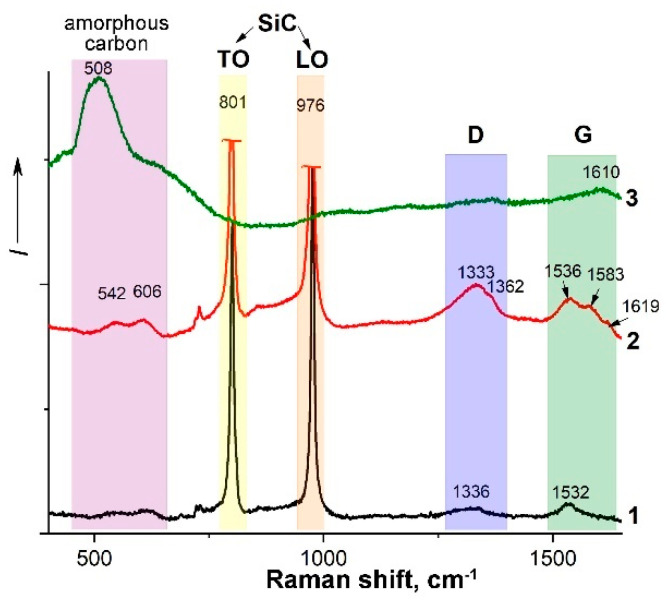
Raman spectra of the surface of ceramic samples ZHS (1), ZHSC (2), as well as the surface of the ZHSC sample after exposure to a supersonic flow of dissociated nitrogen (3).

The study of the obtained materials using Raman spectroscopy made it possible to establish (Figure 3) that for both ZHS and ZHSC, intense modes of cubic silicon carbide with maxima at 801 (TO) and 976 cm^−1^ (LO) are observed, while ZrB_2_ and HfB_2_ are inactive in the Raman spectra. Some shifts in the position of the modes to the region of large shifts, with respect to the values 784–804 (for TO) and 968–975 cm^−1^ available in the literature [44,69,70,71], may be due to the high compressive stress concentrated in the ceramic on the SiC grains [72,73]. For the ZHS sample, the presence of some amount of impurity carbon is shown, which is expressed in the presence of two low-intensity broadened bands with maximums in the 540–550 and 600–610 cm^−1^ region, associated with amorphous carbon, as well as D- and G-peaks. The introduction of multilayer CNTs into the ZHSC sample resulted in the appearance of their characteristic peaks with maxima in the range of 1351–1361 (D-mode) and 1578–1594 cm^−1^ (G-mode) [39,40,44,45,46,74,75], overlapping with those characteristics of the impurity amorphized carbon remaining after the SiC carbothermic synthesis. The observed local maximum at 1619 cm^−1^ may be related to the presence of defective sp^2^ carbon atoms at the ends of the aggregates of multilayer nanotubes.

**Figure 4:**
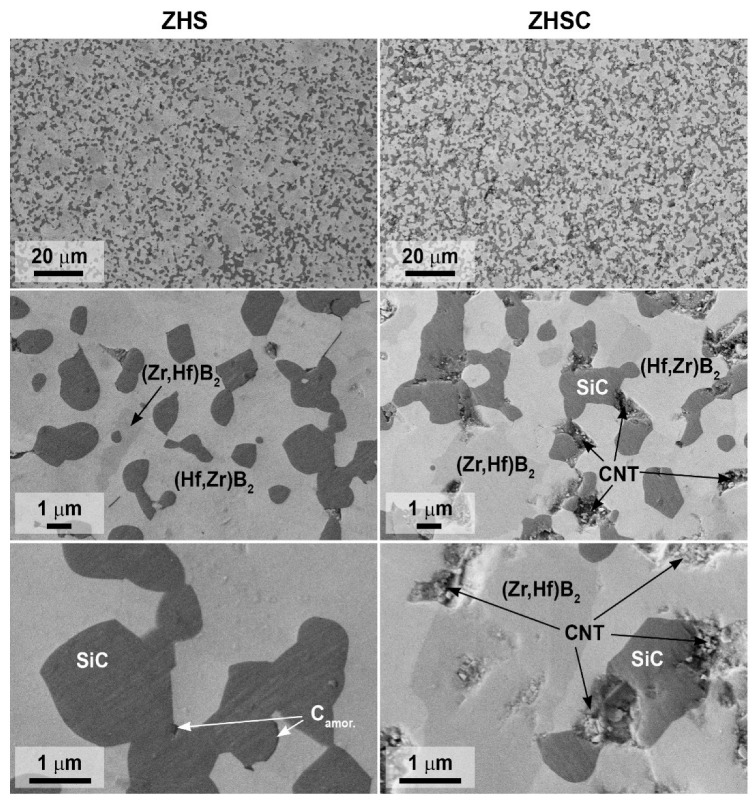
Microstructure of ZHS and ZHSC samples based on SEM data (based on secondary electron detector data), accelerating voltage 2 kV.

**Figure 5:**
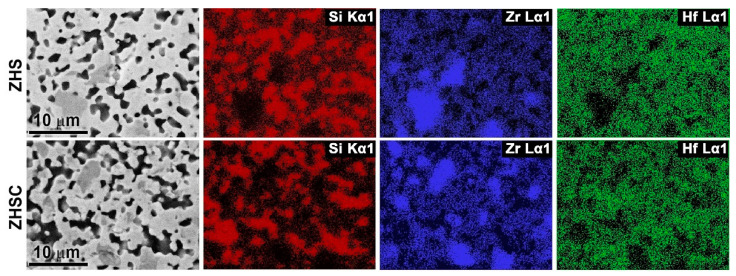
Mapping the distribution of elements Si, Zr, and Hf on thin sections of samples ZHS and ZHSC (EDX).

SEM of thin sections of ZHS and ZHSC samples, performed at an accelerating voltage of 2 kV to increase the contrast, showed that the grain size of the boride phase was 2–3 µm, and silicon carbide–~1–1.5 µm (Figure 4). At the same time, relatively large aggregates of the zirconium–hafnium diboride phase up to 5–10 μm in size were found for the ZHS sample. In this case, as evidenced by the mapping of Si, Zr, and Hf element distributions (Figure 5), the core of such formations is a ZrB_2_-based solid solution. 

In other areas where silicon is absent, the presence of zirconium and hafnium is simultaneously observed, i.e., a solid solution based on the HfB_2_ phase is formed. In addition, the ZHS sample is characterized by the distribution of excessively introduced amorphous carbon (formed during the pyrolysis of phenol-formaldehyde resin and necessary for the synthesis of silicon carbide) at the boundary between the ceramic grains (Figure 4, the darkest areas). The peculiarity of the ZHSC sample microstructure is that large areas of metal diborides localization are much rarer, there is also a solid solution based on the ZrB_2_ phase in their core (Figure 5). For this sample, the formation of aggregates, which constitutes intrinsic inclusions of 1–2 μm in size, typical of multilayer nanotubes, is observed. It should be noted that the microphotographs show the fracture pattern typical of the materials modified with weakly disaggregated CNTs: they are pulled out of the ceramic material (Figure 4). The data of EDX analysis in the area of 19 × 29 microns show that for both ZHS and ZHSC samples, the molar ratio n(Zr):n(Hf) is 1.0 ± 0.2, which corresponds to the specified value of 0.9 within the error of the method.

### 3.2. The Study of the Behaviour of ZHSC Ceramic Material under the Influence of the Supersonic Flow of Dissociated Nitrogen

Earlier, the authors studied the behaviour of the ceramic material HfB_2_-30 vol.%SiC and the sample modified with 2 vol.% graphene (HfB_2_-SiC-C_G_), when heated in underexpanded jets of a dissociated nitrogen high-frequency plasmatron VGU-4 [54]. It was found that at the heat flux density in the range of 244–290 W·cm^–2^, both samples acquired a surface temperature of ~2000 °C and significantly changed their chemical composition: a hafnium carbonitride phase appeared and significantly decreased the content of silicon carbide. Whether these processes are primarily caused by the increased surface temperature or by the chemical activity of the nitrogen atom, must be determined by further experiments.

**Figure 6:**
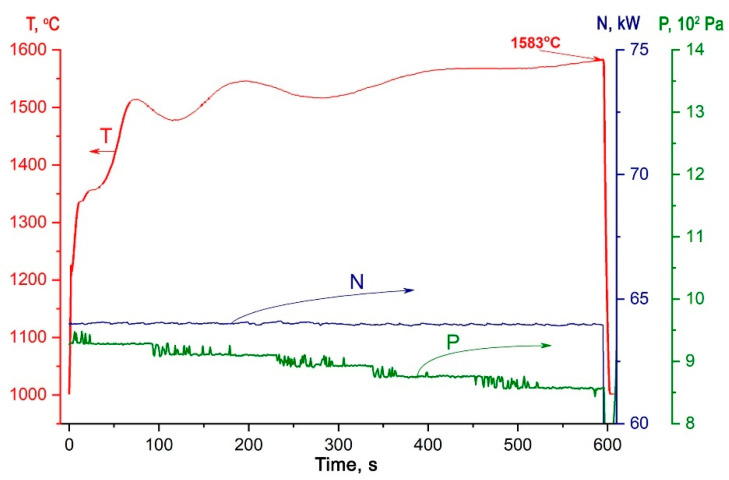
Variation of the color temperature in the center of the sample face (T), plasmatron generator power by anode supply (N) and pressure in the pressure chamber (P) in the experiment on heat exchange of ZHSC ceramics in an underexpanded high-enthalpy nitrogen jet.

To study the thermochemical effects, the CNT-modified ZHSC sample fixed in a water-cooled model was injected into a high-enthalpy flow of dissociated nitrogen at steady-state parameters: N = 64 kW, pressure to the plasmatron barochamber 8.6–9.3 × 10^2^ Pa. The change in the average surface temperature as the experiment proceeds, obtained using an infrared pyrometer, is shown in Figure 6. It is seen that during the first minute of exposure, the temperature increases from 1288 to 1431 °C, and then it cyclically changes smoothly in the temperature range of 1500–1580 °C with a tendency to increase that may be associated with chemical processes occurring on the surface and periodic carry-over of reaction products. The maximum average temperature was 1583 °C at the end of the 10th minute.

**Figure 7:**
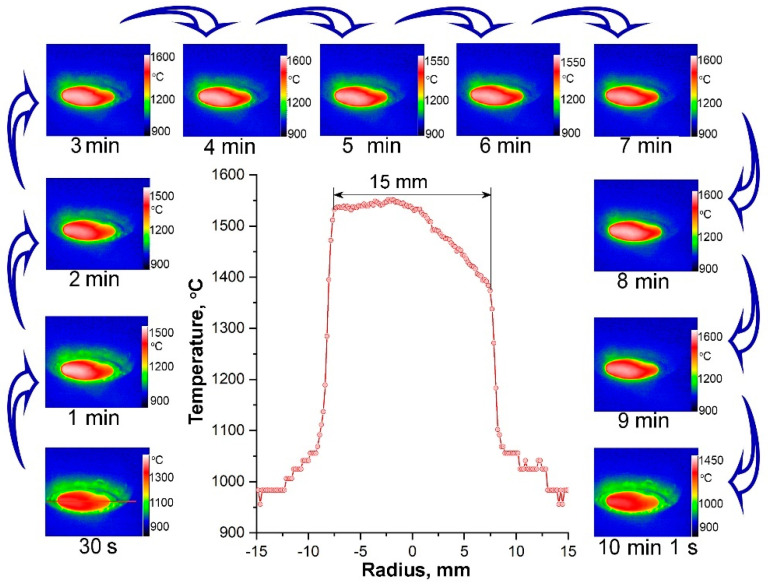
The thermal images at specific moments of the experiment and typical temperature distribution over the diameter of the ZHSC sample (300 s).

The study of temperature distribution over the sample surface using a thermovisor (Figure 7) showed that the observed relatively low average temperature was associated with heat leakage into the water-cooled model due to lateral contact. This also determines a significant asymmetry of temperature distribution along the diameter–a temperature difference of ~170–380 °C. is observed. Additional evidence of some heat leakage into the model is a slightly decreased value of the heat flux measured just before the end of the experiment, which was 206 W·cm^−2^. 

Evaluation of the integral radiation coefficient εt showed that with exposure and, accordingly, changes in the chemical composition and surface roughness of ZHSC ceramics, this indicator increased from 0.68 (30 s) to 0.88 (600 s).

At the end of the heating, the pressure flow into the plasma chamber was room air with the cooled sample, i.e., during the cooling of the sample, its partial oxidation is possible.

The mass loss was 0.001 g (0.03%). The geometric size of the sample did not change. The front surface predominantly acquired a velvety black colour, less intense on the side from which, presumably, the contact of the sample with the water-cooled model was realized. 

X-ray diffraction (XRD) of the surface (Figure 2, X-ray pattern 3) indicates a significant change in the phase composition. In particular, besides the reflexes of the main solid solution phase (Hf,Zr)B_2_ and silicon carbide, there are reflections of monoclinic zirconium oxide, which probably appeared during the sample cooling, as well as a new cubic zirconium–hafnium carbonitride phase (the lattice parameter is intermediate between those of Hf_1_Zr_1_N_2_ [76] and Zr_2_C_1_N_1_ [77] phases). At the same time, there is a change in the composition of the solid solution of zirconium–hafnium diboride in the near-surface layers, subjected to thermal and chemical effects: the reflex position indicates an even greater content of hafnium than that noted for the original sample, and the asymmetric shape with local maxima indicates a possible predominant interaction of atomic nitrogen is with ZrB_2_ (with the formation of the nitride phase). A slight shift in the position of the low-intensity reflex at 2θ = 26.5–27.0° may indicate the appearance of a strongly amorphized boron nitride BN [78]. 

An analysis of Raman spectra of the ZHSC sample surface after thermochemical treatment (Figure 3, spectrum 3) showed that there was no silicon carbide directly on the surface, but there was a broad and intense mode with a maximum at ~500–520 cm^−1^, which can be attributed to amorphous carbon. 

**Figure 8:**
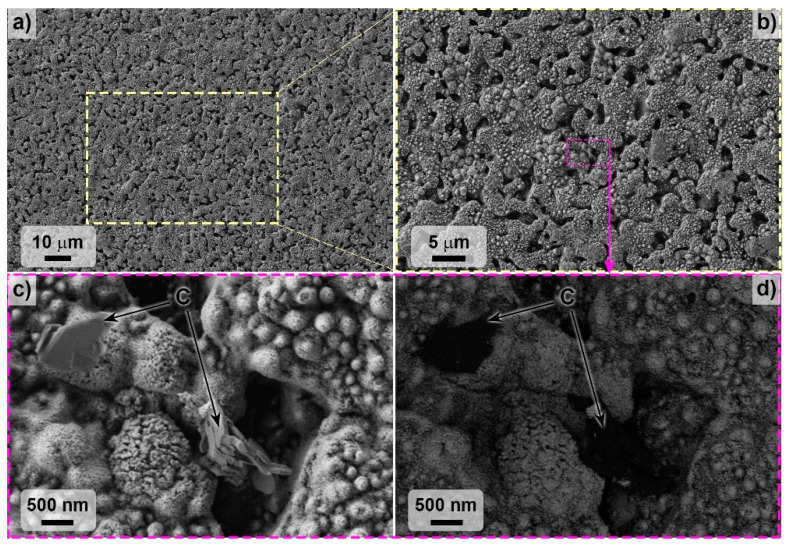
Microstructure of ZHSC sample surface after thermochemical treatment; SEM, accelerating voltage 1 kV: (**a**–**c**)—according to secondary electron detector data, (**d**)—in contrast mode by mean atomic number.

**Figure 9:**
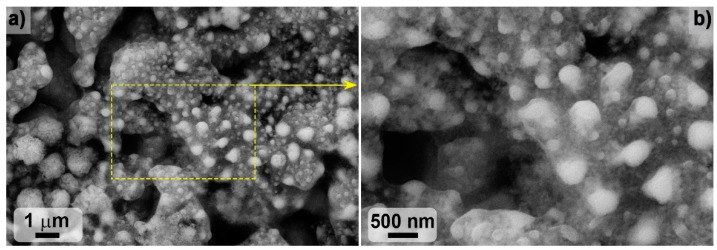
(**a**) ZHSC surface microstructure after thermochemical treatment; (**b**) SEM, accelerating voltage 20 kV.

The SEM microstructure of the ZHSC sample surface after the experiment (Figure 8 and Figure 9) showed that the result was the formation of bulgings with a heterogeneous structure. As can be seen from Figure 8, in some areas, flat carbon aggregates are observed on the surface, probably formed by the consolidation of large agglomerates of multilayer CNTs in the volume of materials. The application of a higher accelerating voltage of 20 kV (Figure 9) showed that the observed bulges consisted of columnar formations of a denser phase with a diameter of 200–500 nm, surrounded by a “coat” of the light phase, presumably, amorphous carbon or boron and silicon nitrides.

**Figure 10:**
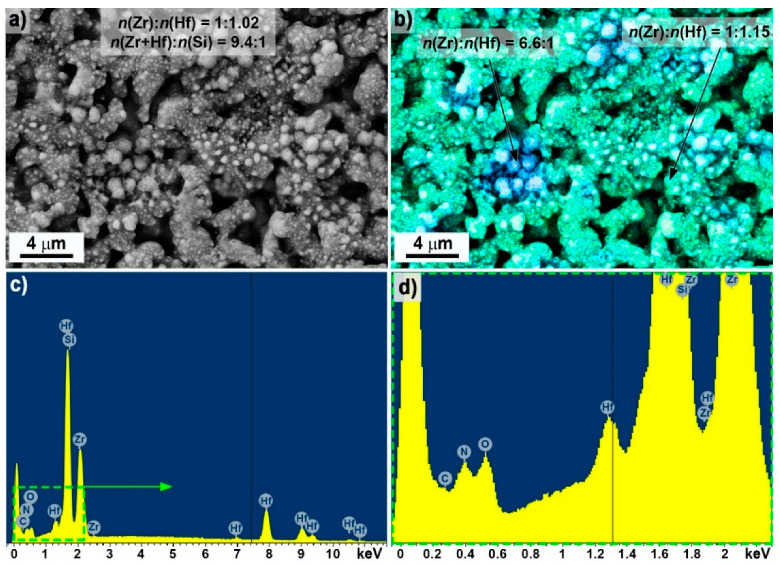
The elemental distribution mapping ((**a**,**b**), zirconium–blue pixels, hafnium–green): X-ray elemental microanalysis data of the entire microphotograph area (**a**) and individual sections (**b**), as well as the corresponding EDX spectrum (**c**), enlarged fragment EDX spectrum (**d**).

The EDX analysis of the 19 × 29 μm region (Figure 10) shows that the molar ratio n(Zr):n(Hf) is close to the specified value of 0.9, while the local distribution analysis shows areas with elevated zirconium content up to n(Zr):n(Hf) = 6.6:1, which are also characterized by elevated oxygen content. One should also note the reduced silicon content relative to the calculated one: n(Zr + Hf):n(Si) is 9.4 compared to 1.6. In addition, despite the high error in determining the light elements, there is an increased content of nitrogen on the surface (Figure 10d), probably as part of the nitrides and carbonitrides of metals, boron and silicon. 

Thus, it was found that the previously observed surface chemical processes for samples of HfB_2_–SiC and HfB_2_–SiC–C_G_ composition under the influence of high-enthalpy nitrogen flows at ~2000 °C [54] take place at a much lower temperature of ~1550–1600 °C.

In order to identify the likely chemical reactions whose products are zirconium/hafnium carbonitride and amorphous carbon found on the surface, the temperature dependencies of ΔG_r_(T) of the interaction processes of ZrB_2_ and SiC with gaseous N_2_(g) and N(g) were calculated.

**2ZrB_2_(c) + 3N_2_(g) = 2ZrN(c) + 4BN(c)** (1)

**ZrB_2_(c) + 3N(g) = ZrN(c) + 2BN(c)** (2)

SiC(c;cub) = Si(g) + C(c;graphite)(3)

2SiC(c;cub) + N_2_(g) = 2Si(g) + 2CN(g) (4)

SiC(c;cub) + N(g) = Si(g) + CN(g)(5)

SiC(c;cub) + N_2_(g) = SiN(g) + CN(g) (6)

**SiC(c;cub) + 2N(g) = SiN(g) + CN(g)** (7)

2SiC(c;cub) + N_2_(g) = 2SiN(g) + 2C(c;graphite) (8)

**SiC(c;cub) + N(g) = SiN(g) + C(c;graphite)** (9)

**3SiC(c;cub) + 4N(g) = Si_3_N_4_(c) + 3C(c;graphite)** (10)

**Figure 11:**
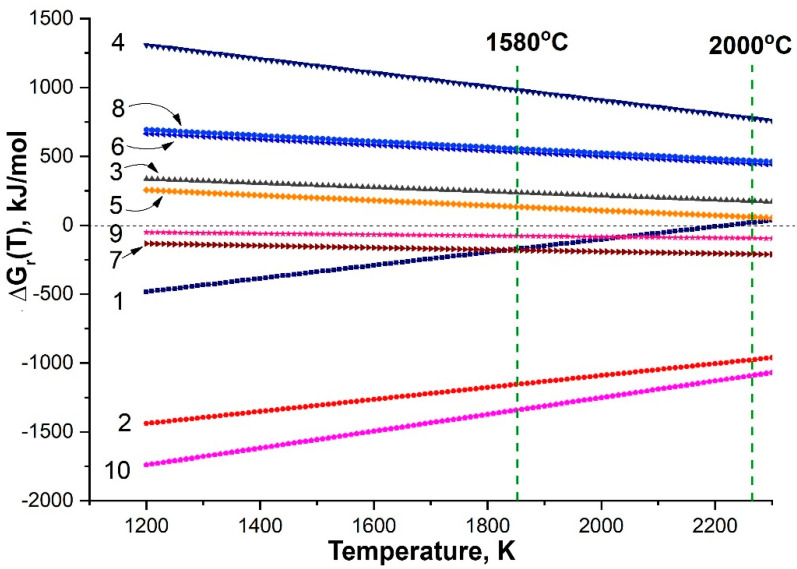
The temperature dependences of ΔG_r_(T), calculated for reactions (1–10) in the temperature range 1200–2300 K using the IVTANTERMO software package with a built-in thermodynamic database; numbers denote reaction numbers.

It was found (Figure 11) that throughout the temperature range that was established on the surface of the ZHSC sample and the previously studied HfB_2_–SiC and HfB_2_–SiC–C_G_ samples [54] (1500–2000 °C), the reaction Gibbs energy was negative for Equations (2), (7), (9) and (10) (arranged as ΔG_r_(T) increases). That is, the formation of carbon is most likely due to the reaction of silicon carbide with atomic nitrogen, as well as zirconium nitride. In this case, the formation of ZrN(c) based on ZrB_2_(c) is so thermodynamically advantageous that at temperatures < 2200 K, this process is allowed even in the interaction with molecular nitrogen (reaction 1).

The absence of Si_3_N_4_ reflexes on the X-ray surface after thermochemical influence (Figure 2, X-ray pattern 3) is possible in case of its X-ray amorphous state or if its formation mechanism is gas-phase and its entrainment by high-speed gas flow occurs. At the same time, a sharp decrease in the silicon content (according to EDX analysis–Figure 10) also draws attention to the possibility of simultaneous reactions 7 and 9, which result in the formation of a gaseous product SiN(g).

## 4. Conclusions

A method of manufacturing dense ceramic materials containing zirconium and hafnium diborides, nanocrystalline silicon carbide and multilayer CNTs, using a combination of sol-gel synthesis and reactive hot-pressing approaches at 1800 °C, has been developed. It was found that the introduction of ZrB_2_–HfB_2_–SiC multilayer nanotubes (10 vol.%) into the UHTC composition led to an increase in sample consolidation efficiency at the final stage (at the temperature > 1600 °C). The obtained ZrB_2_–HfB_2_–SiC and ZrB_2_–HfB_2_–SiC–C_CNT_ materials were characterized by a complex of physical and chemical analysis methods. It was found that under these conditions of reaction hot pressing, the formation of predominantly solid solution Zr_0.31(4)_Hf_0.69(4)_B_2_, took place, but the form of reflexes indicated the presence of a second solid solution based on the zirconium diboride phase. 

The study of the effects of exposure of the modified ZrB_2_–HfB_2_–SiC–C_CNT_ sample to a high-speed flow of partially dissociated nitrogen using a high-frequency plasmatron showed that due to the features of sample installation in the water-cooled model (touching the side surface) and the corresponding heat outflow into the model, the average surface temperature of the experiment did not exceed 1583 °C. Nevertheless, and in this case, as it was noted earlier for samples of close composition, a significant change in the chemical composition and surface microstructure was observed. Thus, XRD showed the presence of cubic zirconium–hafnium carbonitride and, probably, amorphized boron nitride in the near-surface layer of the sample. The surface appearance after thermochemical exposure and Raman spectroscopy indicates the presence of amorphous carbon on the surface. As a result of the evaluation of the integral emission coefficient ε_t_, it has been established that the supersonic flow of dissociated nitrogen results in an increase from 0.68 to 0.88.

Thus, the experiment showed that despite the low values of mass loss of ultrahigh-temperature ceramics ZrB_2_–HfB_2_–SiC–C_CNT_, under the influence of the supersonic flow of underexpanded high-enthalpy nitrogen jets, it was characterized by a significant change in the chemical composition, which accordingly caused a change in the radiation coefficient and the catalytic surface.

## Data Availability

The raw/processed data required to reproduce these findings cannot be shared at this time as the data also forms part of an ongoing study.
